# Therapeutic targeting of ocular diseases with emphasis on PI3K/Akt, and OPRL pathways by *Hedera helix* L. saponins: a new approach for the treatment of *Pseudomonas aeruginosa*-induced bacterial keratitis

**DOI:** 10.1007/s13659-025-00514-x

**Published:** 2025-05-12

**Authors:** Sherif A. Hamdy, Shymaa Hatem, Heba Elosaily, Abrar Gomaa Abd-Elfattah Hassan, Rana Elshimy, Ahmed H. Osman, Riham A. El-Shiekh

**Affiliations:** 1 Pharmacognosy Department, Faculty of Pharmacy, Cairo University, Kasr El-Aini Street, Cairo, 11562 Egypt; 2 Department of Pharmaceutics and Pharmaceutical Technology, Faculty of Pharmacy, Future University in Egypt, New Cairo, Egypt; 3 Biochemistry Department, Faculty of Pharmacy, Ahram Canadian University, 4th Industrial Region, 6th of October City, 12585 Giza Egypt; 4 Biochemistry and Molecular Biology Department, Faculty of Pharmacy (Girls), Al-Azhar University, Cairo, 11765 Egypt; 5 Department of Microbiology and Immunology, Faculty of Pharmacy, Ahram Canadian University, Giza, Egypt; 6 Department of Microbiology and Immunology, Egyptian Drug Authority, Cairo, Egypt; 7 Department of Pathology, Faculty of Veterinary Medicine, Cairo University, Giza, Egypt

**Keywords:** *Hedera helix* L., Saponins, α-Hederin, Hedracoside C, Pseudomonas keratitis, Eye disease

## Abstract

*Pseudomonas aeruginosa*-induced bacterial keratitis is one of the most sight-threatening corneal infections associated with intense ocular inflammatory reactions that may lead to vision loss. Hence, this study investigated the efficacy of three nanocomposite chitosan-coated penetration enhancer vesicles (PEVs) to augment the ocular delivery of saponin(s), α-hederin (PEVI), hederacoside C (PEVII), or both (PEVIII) for treatment of *Pseudomonas* keratitis and its induced inflammatory response. The three formulations were prepared using the ethanol injection method and comprehensively characterized. In vitro*,* the antibacterial activity of the three formulations against *P. aeruginosa* was evaluated using agar well-diffusion method, pyocyanin production inhibition, and swarming and twitching motility inhibition assays. The therapeutic effect of the three formulations has been investigated in *P*. *aeruginosa* keratitis by gross lesion monitoring, determination of bacterial bioburden, biochemical markers, histopathological examination, and scoring after 7 days of topical treatment. Data revealed that PEVI, PEVII, and PEVIII nanocomposites showed particle size in the nanometer range, high entrapment efficiency, good stability, and sustained release of the saponins throughout 24 h. Among them, PEVIII exhibited notably strong in vitro antipseudomonal activity. Additionally, animals treated topically with PEVIII showed an appreciable gross lesion reduction, corneal tissue improvement, and formidable bacterial load reduction compared with untreated and gentamicin sulfate eye (GENTAWISE^®^) ointment-treated groups. Moreover, PEVIII treatment showed the most significant reduction in TNF-α, NF-κB, ROS levels, and OPRL virulence gene expression while enhancing PI3K/Akt activation. Therefore, this study offers PEVIII as a promising treatment for *P. aeruginosa* keratitis.

**Graphical Abstract:**

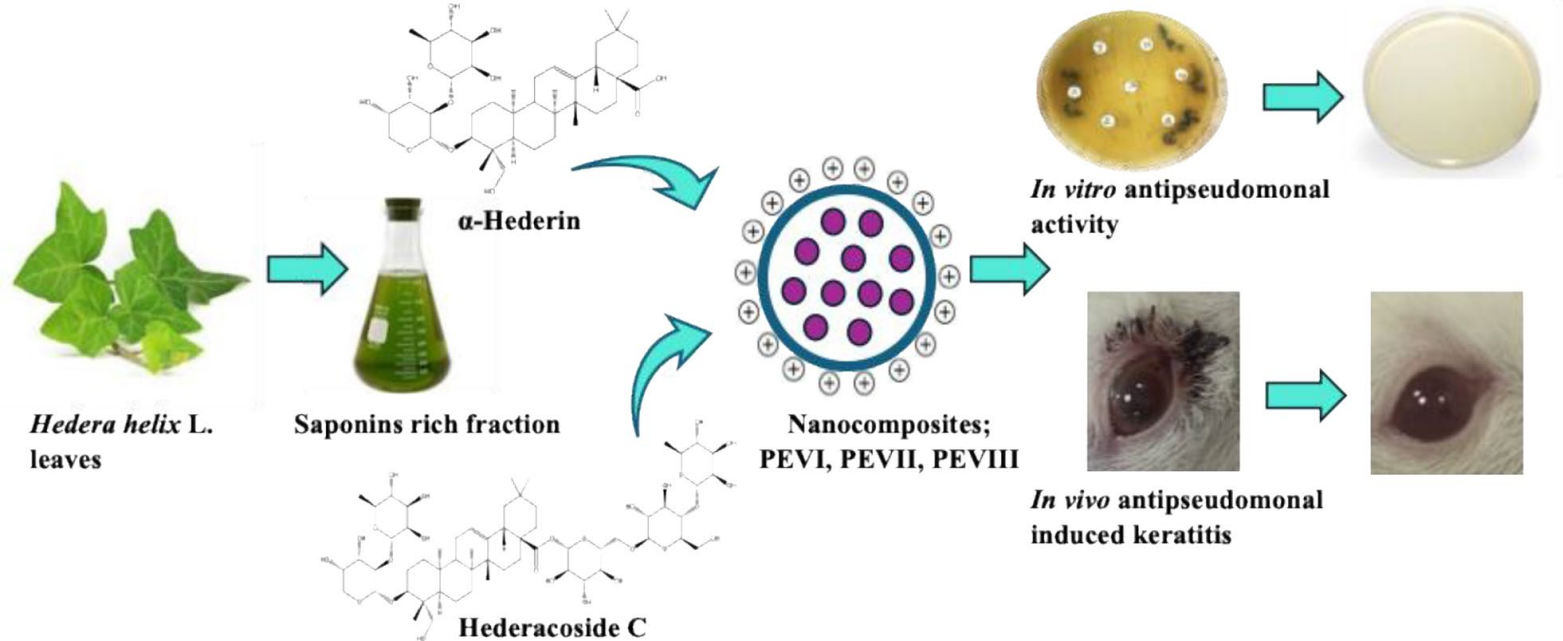

**Supplementary Information:**

The online version contains supplementary material available at 10.1007/s13659-025-00514-x.

## Introduction

Bacterial keratitis (corneal infection) is an overwhelming ailment responsible for two million cases yearly. *Pseudomonas aeruginosa* belongs to ESKAPE pathogens and is considered the most common causative agent of bacterial keratitis, representing a major healthcare problem worldwide [1, 2]. Pseudomonal corneal ulceration and keratitis represent severe and therapeutically challenging ocular pathologies, necessitating a meticulous treatment regimen comprising antimicrobial and anti-inflammatory agents to overcome the risk of irreversible blindness [3, 4].

Yet, hospitalization is obligatory for some cases, like immune-compromised patients with such infections. *P. aeruginosa* is widely acquiring resistance to different classes of topical broad-spectrum antimicrobials [5], including carbapenem antibiotics, such as imipenem and meropenem, in clinical settings, which is a frightening obstacle in the treatment protocol of *P. aeruginosa* [6]*.* Furthermore, *P. aeruginosa* has a wide panel of virulence traits, including twitching and swarming motility, pyocyanin pigment production, and different virulence genes that aid in bacterial invasion and toxicity during infections [5]. Twitching motility via bacterial pilli is a surface-associated motility not required for initial bacterial adherence but has a key role in tissue penetration or bacterial dissemination [7]. Meanwhile, pyocyanin (1-hydroxy-5-methyl-phenazine) is a blue-green pigment produced only by *P. aeruginosa* strains, which augments the virulence of the bacterium and produces reactive oxygen species (ROS), leading to tissue damage, proliferation, and blindness [7]. Additionally, the oprL gene (Encoding membrane lipoprotein L) is predominantly involved in *P. aeruginosa*-related infections and is crucial for maintaining bacterial integrity and protecting against oxidative stress. Although OprL serves as a marker for identifying the genus *Pseudomonas*, it specifically detects *P. aeruginosa* [8]. These two issues complicate the treatment process caused by this pathogenic bacterium. Following the invasion of the bacterial pathogens into the corneal tissue, the host's innate immune response is triggered, initiating a cascade of defense mechanisms aimed at pathogen clearance. Several molecular pathways could regulate the host’s response to *P. aeruginosa* infection. For example, the PI3K/Akt signaling pathway is crucial in modulating inflammation, apoptosis, and proliferation in corneal epithelial cells (CECs) [9]. Besides, reactive oxygen species (ROS) also participate in the regulation of apoptosis and inflammation in CECs in a concentration-dependent manner. For instance, high concentrations of ROS can suppress the PI3K/Akt pathway, thereby promoting apoptosis and inflammation. Conversely, low to moderate ROS levels activate the PI3K/Akt pathway, fostering CEC proliferation and inhibiting apoptotic processes. Nevertheless, uncontrolled PI3K/Akt stimulation can cause inflammation in different circumstances [10]. Several studies validated the contribution of NF-κB to systemic inflammation and keratitis, linking its activation to elevated TNF-α levels and heightened inflammatory responses. For instance, a higher level of the pro-inflammatory cytokine TNF-α was detected in rat models with keratitis, which facilitates inflammatory responses and enhances the activation of NF-κB [11, 12]. Dong et al. [13] reported that persistent NF-κB activation in mice led to severe systemic inflammation and premature mortality [13]. Thus, to lessen the corneal damage caused by *P. aeruginosa* infection, it may be possible to identify therapy targets by comprehending these pathways.

*Hedera helix* L., commonly referred to as common ivy, is a woody species in the Araliaceae family [14, 15]. Historically, ivy leaf extracts have been employed to treat inflammatory bronchial diseases, inflammation, burns, cough, neuralgia, and rheumatism [16, 17]. The leaves and fruits of *H. helix* were also used in Europe to treat gastrointestinal tract-related diseases [18]. Experimentally, ivy extract exhibited antibacterial, antihelmintic, leishmanicidal, and antifungal properties [19]. Moreover, *H. helix* leaf extract has been approved by the German Commission E for its efficacy against chronic inflammatory bronchial conditions and productive coughs due to its expectorant, bronchodilator, antibacterial, and spasmolytic activities. These effects are partly attributed to its triterpene saponins content, particularly hederacoside C, a key active ingredient and marker of *H. helix* leaf extract [14, 15]. Several compounds have been isolated from ivy leaves, including α-hederin, hederacoside-C, hederacolchiside-E, and hederacolchiside-F [16, 17], of which α-hederin and hederacoside C have gained considerable attention for their diverse pharmacological activities, including anti-inflammatory, antioxidant, antimicrobial, and anticancer effects [20–22].

Nanotechnology has significantly advanced the field of ocular drug delivery by introducing innovative therapeutic methods that surpass traditional formulations [23]. These advancements enable the penetration of ocular barriers, enhance transcorneal drug permeability, extend drug residence time, minimize degradation, reduce dosing frequency, improve patient adherence, provide controlled and sustained release, and enable precise drug targeting [24, 25]. Therefore, this study utilized and evaluated three novel hybrid nano-vesicular systems: chitosan-coated PEVs containing two isolated ivy saponins: α-hederin (PEVI), hederacoside-C (PEVII), and a combination of both saponins (PEVIII). These systems were investigated for their impact on the complex molecular pathways regulating the immune response to *P. aeruginosa* keratitis, aiming to identify potential therapeutic targets for mitigating corneal damage and guarding against the emergence of antibiotic resistance.

## Materials and methods

### Instruments

NMR spectra were recorded at 400 (^1^H) and 100 MHz (^13^C) on a Bruker NMR spectrometer, Japan. The NMR spectra were recorded in deuterated CD_3_OD using TMS as an internal standard.

### Isolation of the compounds

Plant material: *Hedera helix* L. leaves were collected from a private garden in Giza, Egypt, and authenticated by Mrs Teresa Labib, Head of the Taxonomists at Orman Botanic Garden. A voucher specimen was deposited in the Herbarium of the Department of Pharmacognosy, College of Pharmacy, Cairo University, Egypt.

Extraction and fractionation: Two kilograms of the air-dried powder were exhaustively extracted with 70% ethanol using a homogenizer-assisted extraction method. The ethanolic extract was combined and evaporated under reduced pressure to dryness. A portion of the extract (200 g) was dissolved in 500 mL methanol and allowed to precipitate for 1 day at 5 °C after the addition of anhydrous acetone (2 L), then filtered. Both filtrate and precipitate were monitored using precoated TLC plates (Fluka, Sigma-Aldrich Chemicals, Germany), where the filtrate was found to be enriched in flavonoids (70 g), and the precipitate was found to be enriched in saponins and further purified by precipitation by acetone to yield saponin-enriched residue (SRF, 130 g). Then, SRF (50 g) was subjected to flash column chromatography on silica gel (0.04–0.063 mm, Merck) and eluted with CHCl_3_–MeOH–H_2_O (26: 14: 3) to afford 5 fractions.

Isolation and identification: Fraction 4 (15 g) was further purified on a polyamide column (SC 6 0.07 mm, Merck) with 50% of MeOH then on another silica gel (0.04–0.063 mm, Merck) and eluted with CHCl_3_–MeOH–H_2_O (26: 14: 3) to obtain 210 mg of compound (1). Compound **1**: Colorless solid; ^1^H NMR (400 MHz, CD_3_OD) and ^13^C NMR (100 MHz, CD_3_OD, TMS) (Supplementary Figs. S1 & S2). Additionally, Fraction 5 (7 g) was purified on a polyamide column (SC 6 0.07 mm, Merck) with 20% of MeOH, then on RP-18 silica gel (Fluka, Sigma-Aldrich Chemicals, Germany) and eluted with MeOH–H_2_O (50: 50) to obtain 140 mg of compound (2). Compound 2: Colorless solid; ^1^H NMR (400 MHz, CD_3_OD) and ^13^C NMR (100 MHz, CD_3_OD, TMS) (Supplementary Figs. S3 & S4).

The structure of saponins was elucidated by ^1^H and ^13^C NMR and compared with the literature to be α-hederin **(1)** and hederacoside C **(2)** [26].

^1^H NMR **(1)** (CD_3_OD, TMS) *δ*: 0.72 (3H, s), 0.84 (3H, s), 0.93 (3H, s), 0.96 (3H, s), 0.97 (3H, s), 0.99 (3H, s), 1.0 (1H, m), 1.17 (3H, s), 1.24 (3H, m), 1.28–1.94 (18H, m), 2.88 (1H dd, J = 13.86, 3.84 Hz), 3.29–3.93 (12H, m), 4.57 (1H, d, *J* = 9.0 Hz), 5.17 (1H, d, *J* = 1.35 Hz), 5.26 (1H, s).

^13^C NMR **(1)** (CD_3_OD, TMS) *δ*: 12.3, 14.9, 16.3, 16.5, 17.4, 22.5, 22.7, 23.1, 25.0, 25.0, 27.4, 30.2, 32.0, 32.4, 33.5, 36.2, 38.2, 39.1, 41.3, 41.5, 42.5, 45.8, 46.2, 46.7, 46.9, 63.3, 63.3, 67.7, 68.7, 70.7, 70.7, 72.2, 72.5, 72.5, 75.3, 80.9, 100.5, 102.9, 122.2, 143.8, 180.8.

^1^H NMR **(2)** (CD_3_OD, TMS) *δ*: 0.72 (3H, s), 0.82 (3H, s), 0.93 (3H, s), 0.97 (3H, s), 1.00 (3H, s), 1.16–2.06 (31H, m), 2.86 (1H dd, *J* = 14.71, 4 Hz), 3.26–4.09 (29H, m), 4.41 (1H, d, *J* = 10.62 Hz), 4.56 (1H, d, *J* = 8.05 Hz), 5.18 (1H, m), 5.27 (1H, m), 5.36 (1H, d, *J* = 8.05 Hz).

^13^C NMR **(2)** (CD_3_OD, TMS) *δ*: 12.4, 15.1, 15.1, 16.5, 16.6, 17.3, 22.7, 22.7, 24.9, 25.0, 27.4, 30.1, 32.1, 33.7, 36.2, 38.2, 39.2, 41.1, 41.7, 42.5, 45.8, 46.6, 46.7, 46.7, 47.0, 47.4, 47.7, 47.8, 48.0, 48.3, 48.3, 60.4, 63.2, 62.5, 65.2, 67.9, 68.2, 68.6, 69.2, 69.5, 70.8, 70.8, 71.1, 72.4, 72.4, 73.6, 75.2, 75.4, 76.2, 78.3, 81.2, 94.5, 100.2, 101.5, 102.7, 102.8, 122.4, 143.5, 176.7.

### Formulation and characterization of the prepared nano delivery systems

Composite nanovesicle material: Epikuron 200 was provided by Cargill Texturizing Solutions, Germany. Transcutol was kindly supplied by Gattefosse Company, France. Phosphotungstic acid, low molecular weight chitosan (50 kDa) with 70% acetylation degree, and dialysis membrane with an average flat width of 33 mm were obtained from Sigma Aldrich Company, USA. Absolute ethanol was purchased from Al-Gomhorea pharmaceutical company, Cairo, Egypt. Nanosep centrifuge tubes equipped with an ultra-filter were obtained from Pall Life Sciences, USA.

Preparation of composite nanovesicles using the ethanol injection method: Composite nano-vesicular systems were formulated using the ethanol injection technique, according to Ang et al*.* [27]. In brief, 5 mL of absolute ethanol comprising 50 mg Epikuron 200 (phospholipid), 25 mg of α-hederin, or hederacoside C, or both were inserted dropwise into 10 mL of a warm 0.6% chitosan acetate buffer solution pH 4.6 encompassing 5% transcutol (acting as a penetration enhancer) using a plastic syringe at an injection rate of 2 mL/min. The dispersions were mechanically stirred at 100 r.p.m. (Lab Tech LMS -1003, Korea) maintained in ambient conditions (25 ℃) till complete evaporation of the employed absolute ethanol. Finally, the obtained dispersions were preserved overnight at refrigeration temperature for further investigations. Table 1 shows the composition of the three vesicular preparations.

**Table 1:** Composition and Characterization of composite nanovesicles

Formula code	Amount of phospholipid (mg)	Type of saponin	Amount of saponin (s) (mg)	Type of PE*	Concentration of PE* (% w/v)	Concentration of chitosan (% w/v)	PS (nm) Mean ± S.D	PDI Mean ± S.D	ζ-potential (mV) Mean ± S.D	EE% Mean ± S.D
PEVI	50	α-Hederin	25	Transcutol	5	0.6	283.6 ± 14.83	0.36 ± 0.014	27.36 ± 5.69	91.58 ± 1.69
PEVII	50	Hederacoside C	25	Transcutol			356.9 ± 16.78	0.41 ± 0.016	28.47 ± 4.32	84.61 ± 2.54
PEVIII	50	α-Hederin and Hederacoside C	25 each	Transcutol			422.5 ± 15.69	0.48 ± 0.034	27.63 ± 3.48	95.98 ± 4.87

^*^PE: Penetration enhancer

Determination of the particle size (P.S.), Polydispersity index (PDI), and zeta potential (ζ-potential): Following appropriate dilution, a Zetasizer (model ZS3600, Malvern Instruments Ltd., Worcestershire, UK) was implemented to assess the aforementioned colloidal properties of the composite vesicles [28].

Determination of entrapment efficiency (EE%): Nanosep centrifugal devices were employed to separate the unentrapped drug from loaded nanoparticles [29]. A volume equivalent to 0.5 mL was transferred to the Nanosep centrifugal devices, then centrifuged in a high-speed cooling centrifuge (SIGMA-3-30KS, Germany) at −4 °C and 3000 rpm for 50 min. After dilution with acetate buffer, pH 6.4, the free drug was measured in the filtrate via a reported HPLC method. Isocratic elution was applied using a mobile phase comprising ammonium acetate (pH 8.5) and acetonitrile 70:30 (v/v) at 1.2 mL min^−1^. Moreover, 1 μl sample volume was injected into a C18 column (Agilent 1290 infinity, Germany) with column effluent being monitored at 220 nm at 30 °C [30], using acetate buffer (pH 6.4) serving as the control solution. The following equation was implemented to compute EE% [31]:

EE% = $\frac{At-Af}{At}$ × 100. Where A_t_ represents the total quantity of saponin(s) being integrated into vesicular formulations, and A_f_ represents the quantity of free saponin(s).

Storage stability study: The P.S., PDI, ζ-potential, and EE% of the three preparations were re-determined after 3 months of storage at 2–8 °C to reflect their physical stability [32].

In vitro release study: The present study was assessed utilizing Hanson Franz-type diffusion apparatus (model 60–301–106, CA, Los Angeles, USA) [33]. The cellulose membranes were loaded between the upper and lower chambers of Franz diffusion cells, and the lower chamber comprised 7.2 mL simulated tear fluid (STF), comprising 2 g NaHCO_3_, 0.08 g CaCl_2,_ and 6.7 g NaCl in 1 L of deionized water, which was agitated at 600 rpm for a period of 24 h at 34 ± 0.5 °C [34]. A specified volume of the three nano vesicular formulations was introduced to the upper (donor) chamber, with the saponin(s) suspensions in acetate buffer (pH 6.4) serving as control. At specified time intervals (0.25, 0.5, 1, 2, 4, 6, 8, 12, and 24 h), a volume equivalent to 2 mL was taken out from the release media and compensated with an equal volume of STF. The amount of released saponin(s) from each formulation was analyzed using a reported HPLC method and measured at ƛmax of 220 nm [35]. In addition, the release data were introduced into several kinetic models to evaluate the mechanism of release of the saponin(s) from the obtained dispersions.

Determination of the surface morphological features of the composite nanosystems employing transmission electron microscope (TEM): The surface morphological features of the formulae were assessed using TEM (JEM—100S, Joel, Tokyo, Japan). Samples were negatively stained imparting 1% phosphotungstic acid and examined under TEM [35].

Sterilization of composite nanosystems by Gamma Irradiation: The three preparations were exposed to gamma radiation at room temperature using a 60 °C radiation source at a dosage rate of 10 kGy/hour. After being subjected to gamma rays, the physicochemical properties of the formulae were assessed, and the variations from non-irradiated samples were compared statistically [36]. Furthermore, a sterility test was performed to assess the sterilization efficiency of the gamma radiation dose delivered to the formulae.

### Antipseudomonal activity

#### Bacterial strain

Three clinical keratitis *P. aeruginosa* isolates collected from tertiary care hospitals in the greater Cairo area were used in this study. Standard microbiological techniques such as Gram staining and oxidase testing were used as an initial identification. Further confirmation was performed using the Vitek^®^ 2 automated system (bioMérieux, Marcyl’Etoile, France) [37]. The antibiotic susceptibility profile of *P. aeruginosa* isolates was evaluated using the agar diffusion method against the most clinically used antimicrobials [37, 38]. Results were construed according to the guidelines of the Clinical and Laboratory Standards Institute (CLSI, 2024). Various antibiotic classes were used, such as penicillins (ampicillin, AMP), carbapenems (imipenem, IPM and meropenem, MRP), third-generation cephalosporins (ceftriaxone, CTR and cefotaxime, CTX), and penicillin-like inhibitors with beta-lactamase inhibitors (amoxicillin-clavulanic AMC). Tests were performed twice for confirmation. According to the World Health Organization (WHO), isolates that were resistant to three or more antibiotic classes were considered multidrug-resistant (MDR).

#### In vitro antimicrobial activity

Agar well diffusion: The antimicrobial activity of α-hederin and hederacoside C was evaluated using the agar well diffusion method. A concentration of 1 × 10^8^ CFU/mL of *P. aeruginosa* suspension was streaked on Muller-Hinton agar plates (Oxoid, UK). Six concentrations (100%, 50%, 25%, 12.5%, 6.25%, 3%, 1.5%, and 0.75%) of the investigated compounds were examined. Then, 50 μl of each previous concentration was added to wells before overnight incubation at 37 °C. Diameters of a zone of inhibition were measured, and the experiments were performed in triplicate [39, 40].

#### In vitro anti-virulent activity

Pyocyanin production inhibition: In a broth containing MIC and another containing sub-MIC, the *P. aeruginosa* isolate was grown overnight at room temperature, then centrifuged, and the obtained supernatant was mixed with chloroform, followed by centrifugation. Finally, 0.2 M HCl was added, followed by centrifugation, and the absorbance of pyocyanin was measured at 520 nm [40].

Swarming and twitching motility inhibition: The medium was prepared by the addition of supplementing 0.5% (*w/v*) casamino acids to Luria–Bertani** (**LB), while the twitching medium consisted of 1.5% LB agar. Before agar solidification, MIC and sub-MIC concentrations of PEVIII were added, and then 2.5 μL of the inoculum was applied to the swarming and twitching agar plates before overnight incubation at room temperature. The migration zones appearing on the agar plates were monitored. All motility experiments were conducted in triplicate [41, 42].

#### In vivo evaluation of anti-pseudomonal activity

Antimicrobial resistance profiling of the obtained strains: An MDR carbapenem-resistant isolate, which showed resistance to four different classes of antibiotics, was used for the in vivo animal model (Fig. S5).

Animals: The study was approved by the Research Ethics Committee (REC) of the Faculty of Pharmacy, Ahram Canadian University, Giza, Egypt, under permit number REC2524. The study adhered strictly to the Guidelines for the Care and Use of Laboratory Animals (National Research Council, 2011). A total of 35 healthy female Sprague–Dawley rats, weighing between 200 and 400 g, were used. All animals were acclimated for 7 days in controlled environmental conditions before the experiment commenced. The rats were randomly assigned to seven groups (n = 5 per group). Group 1 served as the negative control; Group 2 was the positive control (infected, non-treated); Groups 3, 4, and 5 were infected and treated with PEVI, PEVII, and PEVIII, respectively; Group 6 was infected and treated with the blank formula; and Group 7 received gentamicin sulphate ophthalmic ointment (GENTAWISE^®^). At the end of the study, the animals were humanely euthanized with ketamine, followed by cervical dislocation.

Induction of keratitis and grouping of animals: A corneal epithelial defect was created in all rats except for negative control groups (group 1) under anesthesia on the first day, and 0.05 mL per rat was inoculated from a solution containing 1 × 10^8^ colony-forming units (CFU)/ mL of *P. aeruginosa* clinical isolate, and those with keratitis at the end of the third day were added to the group. On the 7th day, which was the end of the study, the rats were sacrificed, their globule structures were removed as a whole, and some were stored in 10% formaldehyde for histopathological examination, whereas the rest were frozen for biochemical parameters evaluation [43].

Gross Lesion Monitoring: The mice were examined at 1, 3, 5, and 7 days postinfection (p.i) to monitor disease progression, and digital images of the mice' corneas were taken.

Determination of bacterial bioburden: Ocular bacterial count was estimated after homogenization of samples in 2 mL of phosphate buffer saline (PBS) followed by serial dilution in PBS (1:10, 1:100, 1:1,000, and 1:10,000), then plating on cetrimide agar plates in triplicate and overnight incubation. To ensure reproducibility, the experiment was conducted in triplicate. All colony counts were expressed as log^10^ CFU per milliliter.

Histopathological examination and scoring: Sacrificed rats were examined microscopically by hematoxylin–eosin (H&E) staining by a pathologist who was unaware of the groups, and the results were evaluated and scored. The eye was placed in the solution immediately after enucleation and trimming. A gauze pad was used to keep the eye submerged. The globe remained in Davidson’s solution for 1–2 days. The eye was then taken out of the solution and placed in 10% paraformaldehyde. Tissue specimens were trimmed off, washed, and dehydrated in ascending grades of alcohol. The dehydrated specimens were then cleared in xylene, embedded in paraffin blocks, and sectioned at 4–6 µm thick. The obtained tissue sections were deparaffinized using xylol and stained using hematoxylin and eosin (H&E) for histopathological examination through the electric light microscope [44]. Davidson's Solution consisted of: glacial acetic acid, 100 mL, 95% ethyl alcohol, 300 mL, 10% neutral buffered formalin, 200 mL, and distilled water, 300 mL. Lesions were scored as follows: (−) for the absence of lesion, ( +) for Mild lesion (+ +) for Moderate lesion (+ + +) for Sever Lesion.

Enzyme-linked immunosorbent assays (ELISA): The eye content of tumor necrosis factor-α (TNF-α; SEA133Ra, Cloud-Clone Corp., USA), nuclear factor kappa B (NF-κB; MBS453975, MyBioSource, CA, USA), reactive oxygen species (ROS; MBS039665, MyBioSource, CA, USA), and AKT1 (AKT1; S-F49321, lifespan Biosciences) were measured in the eye homogenate using the corresponding ELISA kit according to the manufacturer’s protocol.

Quantitative real-time PCR (RT-PCR) analysis: The gene expression of PI3K and OPRL was assessed in eye tissues after the extraction of RNA. The Direct-zol™ RNA MiniPrep Plus kit (Cat # R2072, ZYMO Research Corp., CA, USA) was used in the extraction process. The Beckman dual spectrophotometer (USA) was used to assess the purity and concentrations of extracted RNA. As directed by the manufacturer, the SuperScript™ IV One-Step RT-PCR kit (Cat # 12594100; Thermo Scientific, MA, USA) was used.

The forward and reverse primers for PI3K were 5'-ACACCACGGTTTGGACTATGG-3' and 5'- GGCTACAGTAGTGGGCTTGG-3', respectively.

For OPRL, the forward primer was 5'-ATGGAAATGCTGAAATTCGGC-3' and the reverse primer was 5'-CTTCTTCAGCTCGACGCGACG-3'.

For GAPDH forward primer was 5'-TGGATTTGGACGCATTGGTC-3' and the reverse primer was 5'-TTTGCACTGGTACGTGTTGAT-3'.

Quantitative real-time PCR reactions were conducted using the Step One™ system (Applied Biosystems, CA, USA). The relative expression levels of PI3K and OPRL were determined according to the Livak and Schmittgen 2 − ΔΔCt method using GAPDH as an internal standard and presented as fold change from control.

Statistical analysis of data: One-way ANOVA followed by Tukey–Kramer post-test was utilized for data statistical analysis. Bonferroni correction and Cohen’s d were also applied to assess multiple comparisons and effect size, respectively. Data were expressed as mean ± SD, using Graphpad Instat software.

## Results

### Characterization of the prepared nanocomposite delivery systems

Determination of the particle size (PS), Polydispersity index (PDI), and zeta potential (ζ-potential): As demonstrated in (Table 1), the PS values of the vesicular dispersions (PEVI, PEVII, and PEVIII) ranged from 282.6 ± 14.83 to 422.5 ± 15.96 nm, reflecting the existence of a remarkable relationship between the size of the vesicles and their structure, with recorded PDI values not transcending 0.5, which is considered satisfactory [45]. The formulations imparted positive charges ranging from + 27.36 ± 5.69 to + 28.47 ± 4.32 mV (Table 1) with insignificant changes between them (P > 0.05), showing the superior stability of the nanoformulations due to the electrostatic repulsive forces between vesicles.

Determination of entrapment efficiency (EE%): As observed from the results of the EE% (Table 1), it was obvious that all the formulae (PEVI-PEVIII) displayed high EE% values ranging from 84.61% ± 2.54 to 95.98% ± 4.87.

Storage stability study: In terms of physical appearance, the three vesicular dispersions remained unchanged after 3 months of storage (data not shown). Upon storage, composite nanoformulations (PEVI-PEVIII) displayed a slight but statistically significant increase in PS and PDI *(P* < *0.0*5), reflecting vesicles' expansion [24]. Regarding surface charge, the three vesicles showed a slight yet significant decrease *(P* < *0.0*5) in ζ-potential values due to the notable increase in the vesicles' PS upon storage [36]. Furthermore, several studies reported their chemical stability, especially when sequestered into nanoparticles, preserving their effectiveness in the treatment of the disease.

In-vitro release study: The study evaluated the three composite vesicular dispersions (PEVI-PEVIII) in comparison to a saponin suspension in acetate buffer (pH 6.4) as a control. The formulations exhibited coherence with Higuchi diffusion kinetics (Table 2), and they were consistent with the findings of Sharma et al. [46]. In addition, fitting the obtained results into the Korsmeyer-Peppas equation demonstrated *n* values of 0.46, 0.47, and 0.45 for PEVI, PEVII, and PEVIII, respectively, confirming the Fickian diffusion of the saponin(s) from the formulations. This was consistent with the outcomes of Lakshmi et al. [47].

**Table 2:** Release kinetics data for the prepared formulations PEVI, PEVII, and PEVIII

Formula code	r^2^ of Zero order	r^2^ of First order	r^2^ of Higuchi model
PEVI	0.835	0.965	0.979
PEVII	0.868	0.956	0.984
PEVIII	0.851	0.943	0.982

**Fig. 1:**
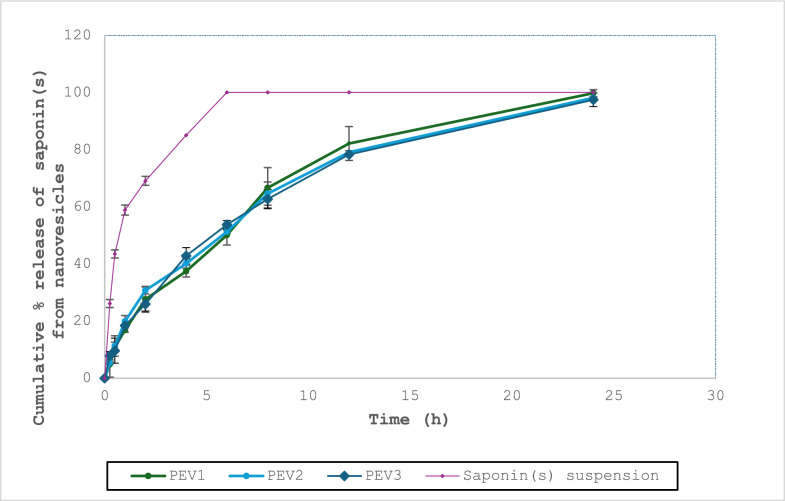
Mean cumulative release of saponin(s) from the formulations (PEVI, PEVII, and PEVIII) compared to control drug solutions

Meanwhile, the three vesicles exhibited a comparable (P > 0.05) sustained release profile of the saponin(s) over a 24 h period compared to the control suspension, in which a rapid release of saponin(s) was observed after 6 h (Fig. 1).

**Fig. 2:**
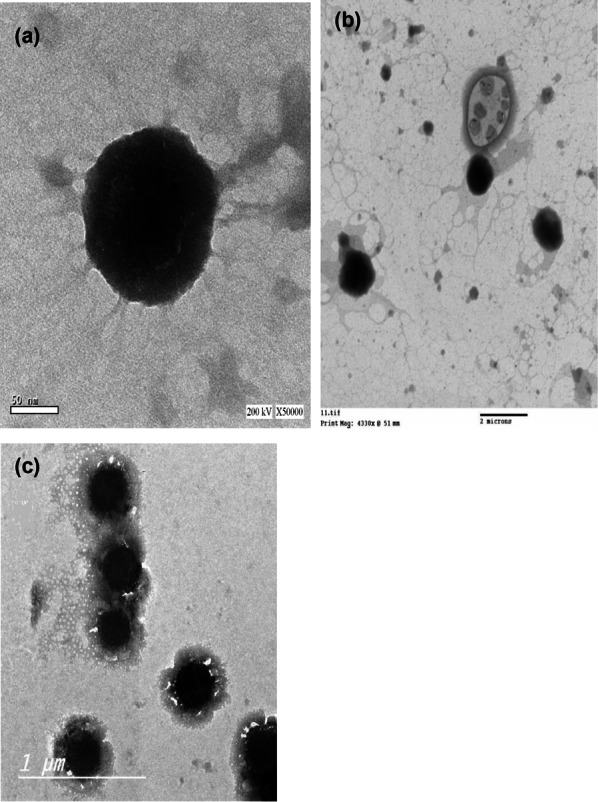
Transmission electron microscope micrographs of the three vesicular systems: **a** PEVI, **b** PEVII, and **c** PEVIII) at; **a** × 14,000 × and **b** × 50,000x

Determination of the surface morphological features of the composite nanosystems employing transmission electron microscope (TEM): The three formulations were selected for photographing using TEM, as displayed in Fig. 2a–c. The photomicrographs revealed well-defined spherical unilamellar nanoparticles with an approximate size of 300–400 nm, which was consistent with the measurements previously obtained using the dynamic light scattering particle size analyzer.

#### Sterilization of composite nanosystems by Gamma irradiation

The three prepared formulations (PEVI-PEVIII) were sterilized using gamma irradiation with a radiation dose of 10 kGy and then examined for microbiological contamination, either fungal or bacterial, to ensure their sterility. After sterilization, P.S., PDI, ζ-potential, and EE% were evaluated again. At the selected radiation dose, all formulations showed no significant changes in their physical properties *(P* > *0.05)* (data not shown). A confirmatory sterility test was performed to determine the sterility of the prepared formulae. The absence of microbiological growth in any of the tested samples confirms the formula's sterility and the effectiveness of gamma irradiation for sterilization at the prescribed level. These findings agreed with those of Abdel Azim et al. [48].

### In vitro antimicrobial activity

MIC values of PEVI, PEVII, and PEVIII were 1024, 512, and 64 μg/mL, respectively.

#### In vitro anti-virulent activity

**Fig. 3:**
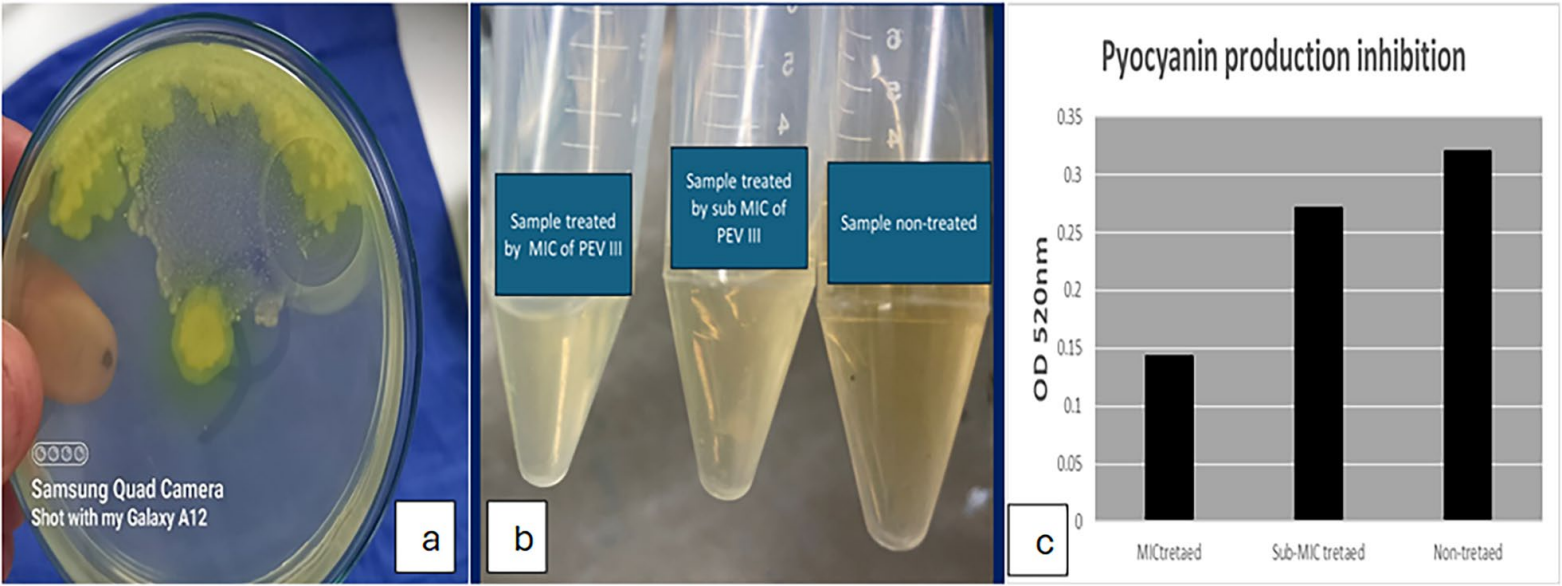
The effect of PEVIII on pyocyanin production: **a** pyocyanin production on cetrimide agar plate, **b** reduction in pigment production, **c** reduction in absorbance

Pyocyanin production inhibition: PEVIII significantly reduced absorbance to 0.143 and 0.2715 upon using MIC and sub-MIC concentrations (Fig. 3).

**Fig. 4:**
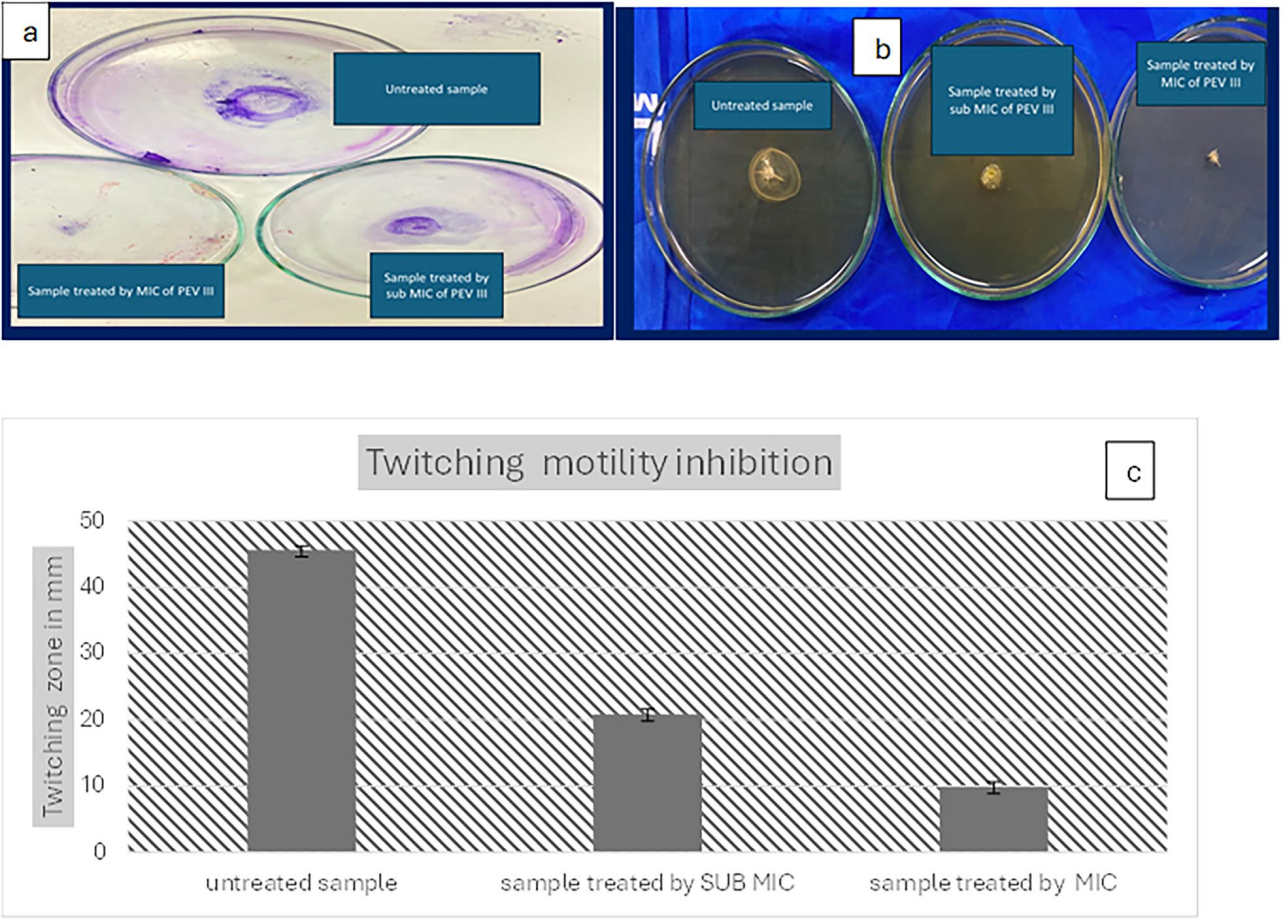
The effect of PEVIII on twitching motility: **a** twitching motility on LB agar plate, **b** twitching motility after CV staining, **c** reduction in twitching zone

**Fig. 5:**
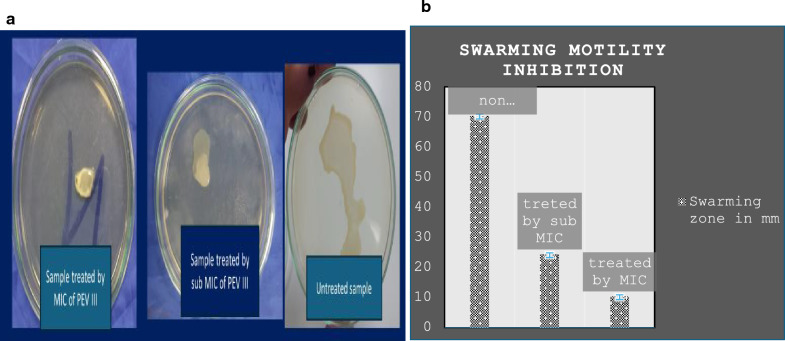
The effect of PEVIII on swarming motility: **a** swarming on LB agar plate, **b** reduction in swarming zone

Twitching and swarming motility inhibition: Both swarming and twitching motility were noticeably reduced with increasing nanocomposite concentration compared to the control (absence of the nanocomposite) (Figs. 4 and 5).

### In vivo evaluation of anti-pseudomonal activity

**Fig. 6:**
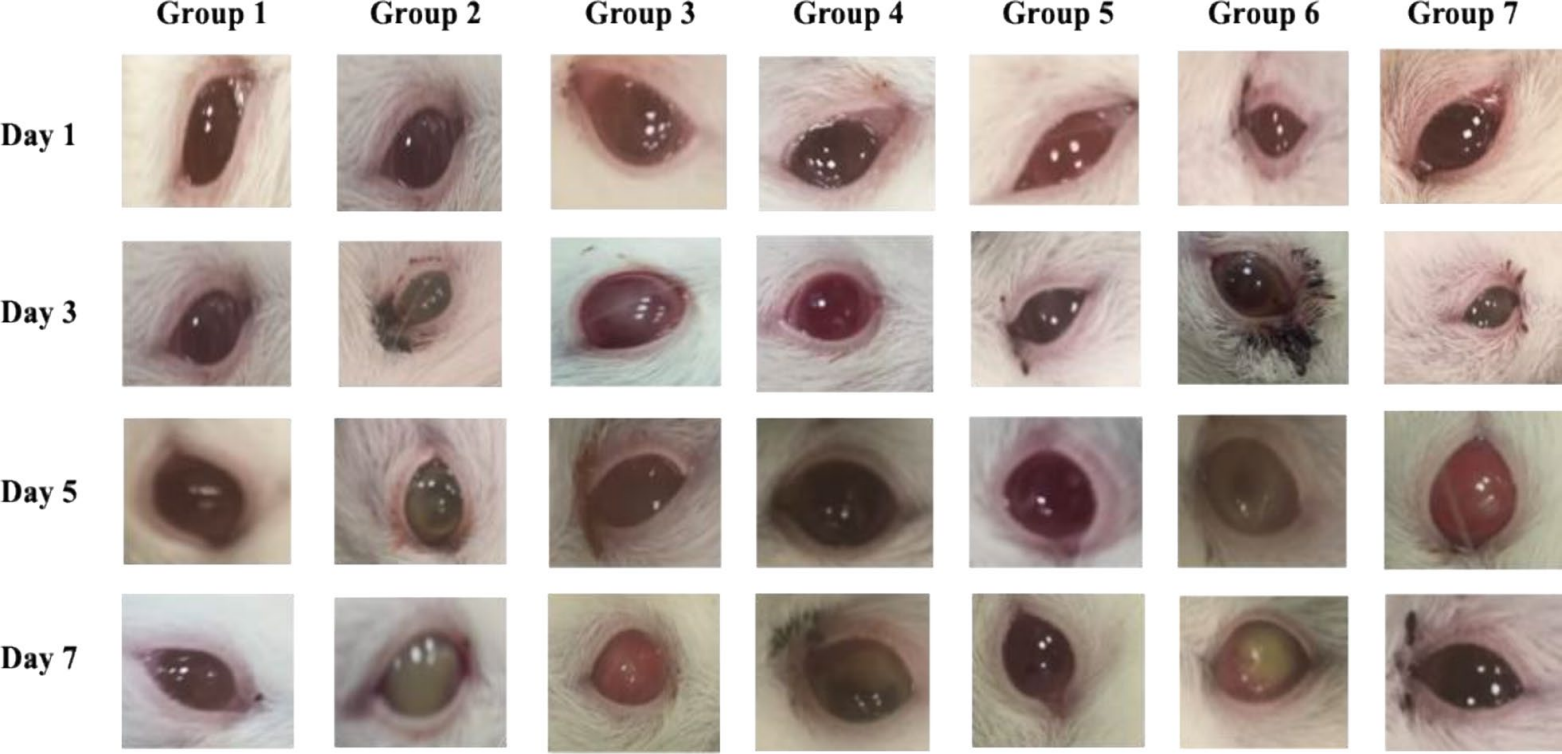
The effect of nanocomposites treatment lesion gross; Group 1) negative control, group 2) positive control infected nontreated, group 3) Infected then treated by PEV I, group 4) Infected then treated by PEVII, group 5) Infected then treated by PEVIII, group 6) Infected then treated by blank formula, group 7) Infected then treated by commercial eye drop (GENTAWISE^®^)

Gross lesion monitoring: The keratitis signs were significantly reduced in the animals treated with PEVIII compared to the control group, as shown in Fig. 6.

Determination of bacterial bioburden: Quantification of the bacterial bioburden in each group over time indicates that the *P. aeruginosa* load in the PEVIII groups is lower than that in the infected non-treated group. In the current study, PEVIII significantly abridged the bacterial load compared with both untreated and gentamicin sulphate eye ointment (GENTAWISE®) commercial-treated groups Table 3.

**Table 3:** The bacterial bioburden of each animal group over time

	Group 1	Group 2	Group 3	Group 4	Group 5	Group 6	Group 7
Day 0	< 10	5 × 10^8^	4.9 × 10^8^	5 × 10^8^	2.4 × 10^8^	3 × 10^8^	3.4 × 10^8^
Day 2	< 10	3.31 × 10^10^	1.8 × 10^9^	3.2 × 10^7^	3.2 × 10^5^	4.5 × 10^9^	4.4 × 10^9^
Day 4	< 10	2 × 10^11^	4.2 × 10^8^	4 × 10^6^	1.7 × 10^2^	1.5 × 10^10^	2.3 × 10^9^
Day 7	< 10	3.5 × 10^12^	2 × 10^6^	2.5 × 10^4^	< 10	2.5 × 10^11^	1.8 × 10^8^

Group 1: negative control, group 2: positive control infected nontreated, group 3: Infected then treated by PEVI, group 4: Infected then treated by PEVII, group 5: Infected then treated by PEVIII, group 6: Infected then treated by blank formula, group 7: Infected then treated by commercial eye drop (GENTAWISE®)

**Fig. 7:**
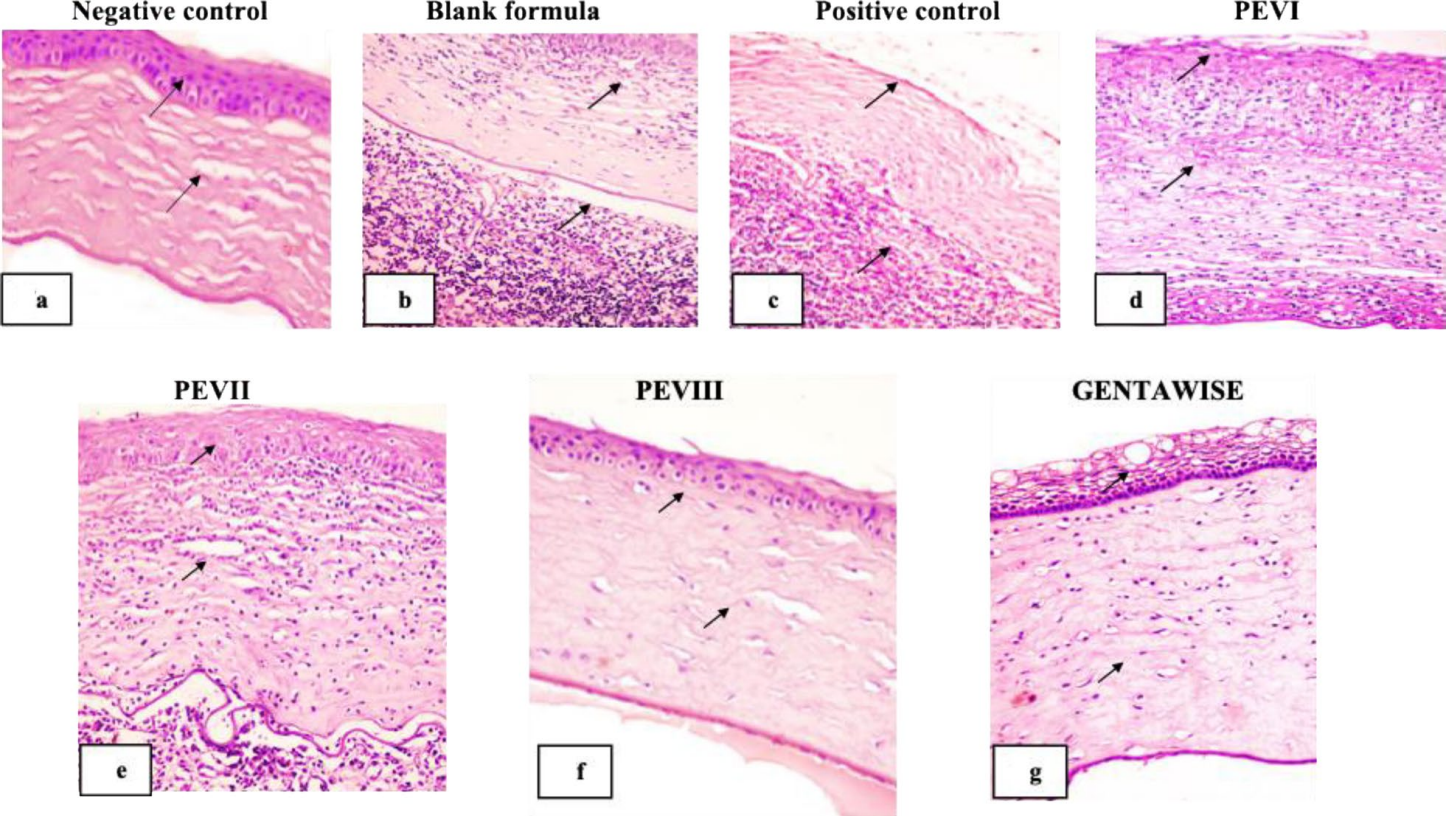
Photomicrograph of corneal tissue section showing: **a** normal histological architecture of corneal epithelium, Bowman’s layer, stroma, Descemet’s membrane arrow: **b**, **c** loss of corneal epithelial lining and massive polymorphonuclear neutrophils infiltration arrow: **d**, **e** epithelial loss neutrophils infiltration, oedema and vascularization of corneal stroma: **f** mild swelling of the outer corneal epithelial lining and oedema of regular arranged collagen bundles arrow: **g** vacuolar degeneration of outer corneal epithelial layers with moderate oedema of collagen bundles arrow (H&E × 200)

Histopathological examination and scoring: Histological sections of the negative control animal group demonstrated normal cornea histological structure, consisting of epithelium, Bowman’s layer, stroma, Descemet’s membrane, and endothelium. Corna was lined by outer non-keratinized stratified squamous epithelium with intact epithelial basal lamina and Bowman’s layer. The corneal stroma appeared as regularly arranged collagen lamellae with intact Descemet’s membrane, which was lined by a single layer of flat endothelial cells Fig. 7a.

The tissue section of the infected eye blank formula & positive control infected non-treated group revealed a disruption of corneal tissue architecture. Loss of the outer epithelial lining of the cornea was seen. Severe corneal damage with intensive polymorphonuclear neutrophil infiltration, degeneration, and necrosis of corneal epithelial layers was also noticed. Disruption of corneal collagen bundles and new vascularization were noticed in the stroma, as shown in Fig. 7b, c.

The tissue section of the infected eye *P. aeruginosa* treated by PEVI or PEVII displayed similar histological pictures. The corneal tissue section showed mild improvement in comparison with the untreated group. Epithelial loss and moderate to severe inflammatory infiltration, mainly neutrophils and macrophages. Oedema and vascularization of corneal stroma were seen in Fig. 7d, e.

The tissue section of the infected eye *P. aeruginosa* treated by PEVIII showed pronounced improvement in comparison with the previous group. Mild swelling of outer corneal epithelial lining with mild edema of regularly arranged collagen bundles without inflammatory cell infiltration Fig. 7f. On the other hand, *P. aeruginosa*-infected eyes treated by GENTAWISE^®^ revealed less improvement than the previous group. Vacuolar degeneration of outer corneal epithelial layers with moderate oedema of collagen bundles was seen in Fig. 7g. The histopathological lesions in different treatments are summarized in Table 4.

**Table 4:** The histopathological lesions in different treatments

Lesions	Negative control	Blank formula	Positive control	PEVI	PEVII	PEVIII	GENTAWISE®
Cornea							
Epithelial loss	−	+ + +	+ + +	+	+	−	+
Inflammatory cell infiltration	−	+ + +	+ + +	+ +	+ +	−	−
Oedema	−	+ + +	+ + +	+ + +	+ + +	+	+ +
Vascularization	−	+ +	+ +	+ +	+ +	−	−

(−) = No lesion ( +) = Mild lesion (+ +) Moderate lesion (+ + +) Sever Lesion

**Fig. 8:**
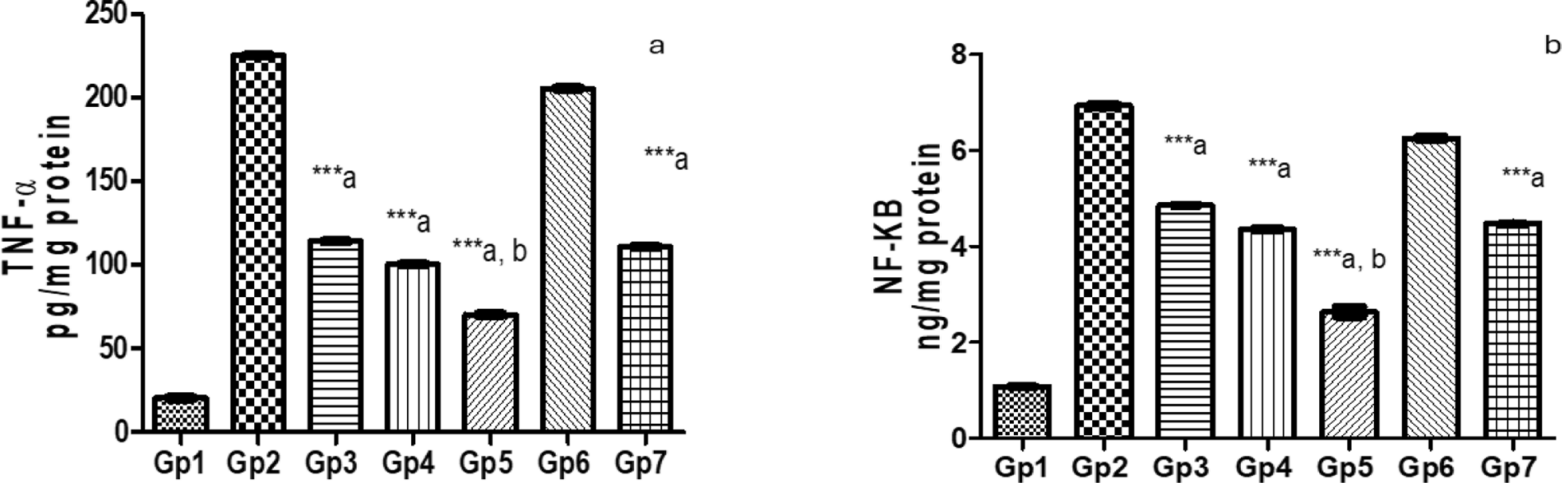
Represents the pro-inflammatory parameters: **a** Tumor necrosis factor-α (TNF-α) and **b** nuclear factor kappa B (NF-κB) of infected mice with keratitis that were treated by different composite nanovesicles with saponins (PEVI, PEVII, PEVIII) (GP3, Gp4, Gp5), respectively, compared to GENTAWISE^®^ treated mice (Gp7). Where the three groups of prepared saponins show significant differences for the three pro-inflammatory parameters from the positive control (Gp2) (*** p ≤ 0.001 denoted by symbol a). PEVIII (Gp5) shows a significant difference also for the two pro-inflammatory parameters from the GENTAWISE^®^-treated group (Gp7) (*** p ≤ 0.001 denoted by symbol b). One-way ANOVA followed by Bonferroni post-hoc test was performed for multiple comparisons. Statistical significance was set at p < 0.05

Comparative efficacy of novel saponin treatments in reducing inflammation in Pseudomonas keratitis: insights against standard therapy: In comparing the treated groups with the Pseudomonas keratitis-infected group, PEVIII treatment was the most effective in reducing inflammation. The PEVI-treated group showed a modest reduction, with TNF-α decreasing by 49.3% and NF-κB by 30%. The PEVII-treated group showed a greater reduction of 55.5% in TNF-α and 37.2% in NF-κB. However, PEVIII treatment had the most significant effect, with a 68.8% and a 62% decrease in TNF-α and NF-κB, respectively, compared to the infected group. On the other hand, GENTAWISE^®^ reduced TNF-α and NF-κB by 50.8% and 35.4%, respectively, highlighting PEVIII as the best therapeutic option. Cohen's d analysis revealed significant effect sizes for the differences in TNF-α and NF-κB levels between the treatment groups and the normal group. The Pseudomonas keratitis-infected group exhibited the largest effect sizes, indicating a substantial increase in both cytokines compared to the normal group. PEVIII-treated mice and GENTAWISE^®^ -treated mice showed moderate to large reductions in both TNF-α and NF-κB levels, suggesting that these treatments effectively reduce inflammation, though not to the baseline levels observed in the normal group (Fig. 8).

**Fig. 9:**
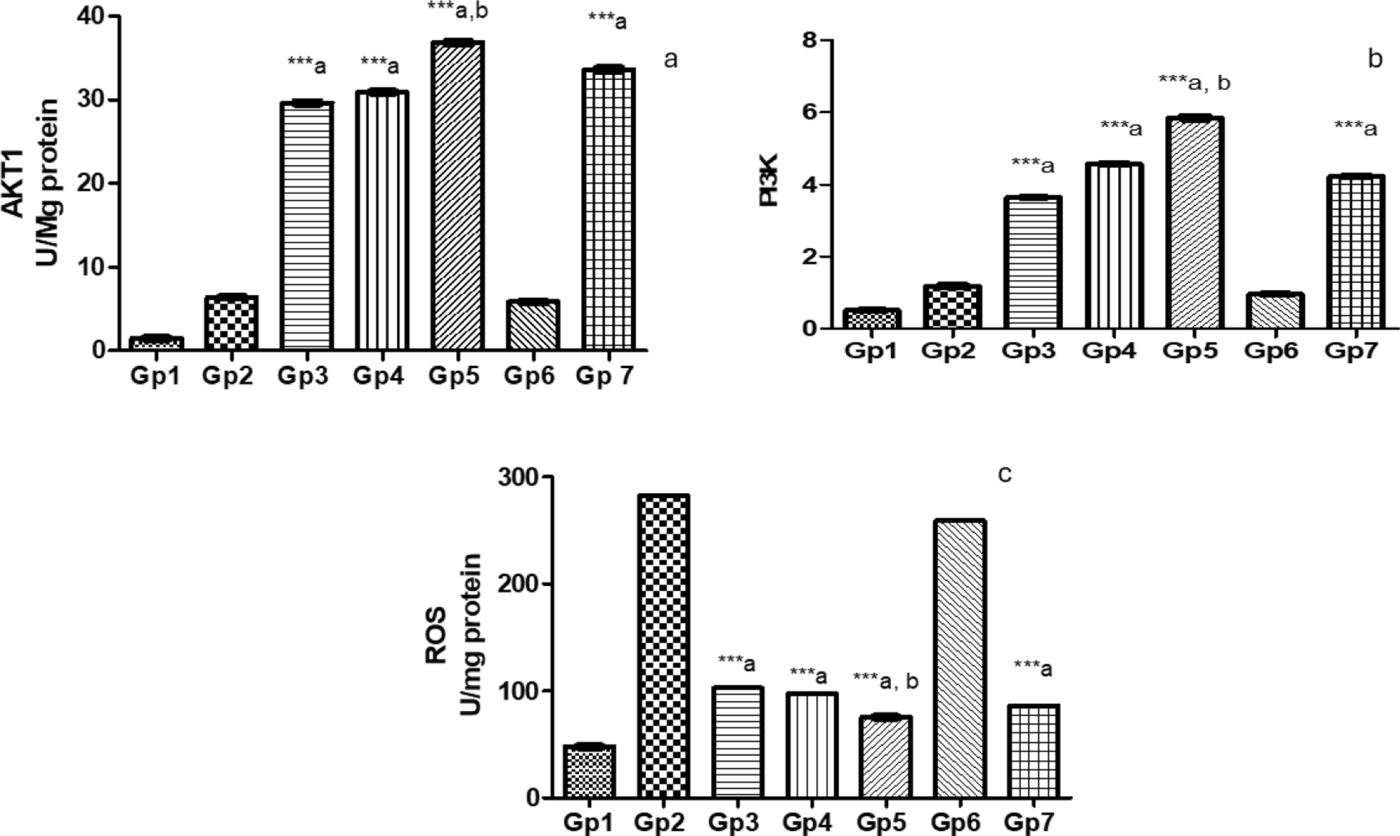
Represents **a** the survival factors: alpha serine/threonine-protein kinase (AKT1) and **b** the gene expression of phosphatidylinositol 3-kinases (PI3K) **c** reactive oxygen species (ROS) of infected mice with keratitis that were treated by different Composite Nanovesicles with Saponins (PEVI, PEVII, PEVIII) (GP3, Gp4, Gp5), respectively, compared to GENTAWISE^®^-treated mice (Gp7). Where the 3 groups of prepared saponins show significant differences for AKT1, PI3K, and ROS levels from the positive control (Gp2) (*** p ≤ 0.001 denoted by symbol a). PEVIII (Gp5) shows a significant difference for AKT1, PI3K, and ROS levels from the GENTAWISE^®^-treated group (Gp7) (*** p ≤ 0.001 denoted by symbol b). One-way ANOVA followed by Bonferroni post-hoc test was performed for multiple comparisons. Statistical significance was set at p < 0.05

Efficacy of novel treatments against Pseudomonas keratitis: a comparative analysis of protective pathway activation and oxidative stress reduction: The efficacy of various treatments was evaluated by comparing AKT1, PI3K, and ROS levels in the treated groups against those in the Pseudomonas keratitis-infected group. The PEVIII-treated group exhibited the highest effectiveness, resulting in an increase in both AKT1 to 5.75-fold and PI3K to 4.97-fold compared to the infected group. Additionally, it demonstrated a significant reduction in ROS levels by 73.3%. In comparison, the GENTAWISE^®^-treated group showed an increase in AKT1 to 5.25-fold and an increase in PI3K to 3.59-fold, while a reduction in ROS by 69.6%. Although the PEVI and PEVII treatments showed improved outcomes, they were less effective than PEVIII and GENTAWISE^®^. Cohen’s d analysis revealed that the PEVIII-treated group had large effect sizes for increased AKT1 and PI3K expression, and a significant reduction in ROS compared to both Pseudomonas infected group and GENTAWISE^®^-treated group (Fig. 9).

**Fig. 10:**
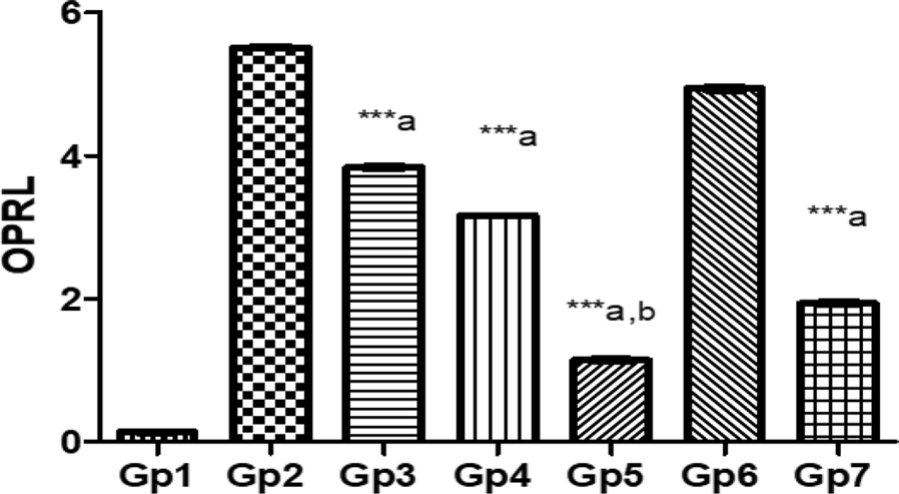
Represents the OPRL expression in infected mice with keratitis that was treated by different composite nanovesicles with saponins (PEVI, PEVII, PEVIII) (Gp3, Gp4, Gp5), respectively, compared to GENTAWISE^®^-treated mice (Gp7). Where the three groups of prepared saponins show significant differences for OPRL expression from the positive control (Gp2) (*** p ≤ 0.001 denoted by symbol a). PEVIII (Gp5) shows a significant difference in the OPRL expression from the GENTAWISE^®^-treated group (Gp7) (*** p ≤ 0.001 denoted by symbol b). One-way ANOVA followed by Bonferroni post-hoc test was performed for multiple comparisons. Statistical significance was set at p < 0.05

The role of OPRL expression in evaluating treatment efficacy for Pseudomonas keratitis: In this study, the effectiveness of various treatments in reducing the virulence factor OPRL in a Pseudomonas keratitis infection model was assessed. Among the treatments, PEVIII was the most effective, leading to a 79.2% reduction in OPRL expression compared to the infected group, highlighting its potential as a strong therapeutic option. GENTAWISE^®^ treatment also significantly reduces OPRL levels, achieving a 64.9% reduction, though it was less effective than PEVIII. PEVII showed a moderate reduction in OPRL expression, with a 42.6% decrease, while PEVI had the smallest effect, reducing OPRL levels by 30.3%. Cohen's d analysis revealed significant effect sizes for the difference in OPRL expression levels between groups (Fig. 10).

## Discussion

To advance the development of novel antimicrobial agents targeting *P. aeruginosa*-induced keratitis, we demonstrated for the first time the antibacterial and anti-inflammatory activities of three nanocomposites, PEVI-PEVIII, against *P. aeruginosa* keratitis in a murine model. The three nanocomposites exhibited high positivity, which could be attributed to the abundance of positively charged amino groups on the surface of the chitosan molecule and the spontaneous formation of densely packed molecules with uniform positive charge [49]. Hederacoside C-containing vesicles (PEVII) exhibited a significantly larger particle size (P.S) compared to α-hederin-containing vesicles (PEVI) (P < 0.05). This difference is attributed to the higher molecular weight of hederacoside C (1221.4 g/mol) relative to α-hederin (751 g/mol), which likely facilitates greater vesicle expansion due to the larger molecular dimensions of hederacoside C. [50]. Co-encapsulation of saponins in PEVIII significantly increased particle size (P.S) compared to individual saponin encapsulation in PEVI or PEVII (P < 0.05), probably due to spatial constraints within the nanosystem or saponin aggregation [51]. The encapsulation efficiency (EE%) of α-hederin-containing vesicles (PEVI) was significantly higher than that of hederacoside C-containing vesicles (PEVII) (P < 0.05), attributable to the higher lipophilicity of α-hederin (Log P 3.6) compared to hederacoside C (Log P -2). This disparity likely results from a greater partitioning of hederacoside C into the aqueous phase relative to α-hederin, as supported by their respective Log P values [52, 53].

PEVIII, incorporating both saponins, demonstrated a notable increase in EE% (P < 0.05), which can be linked to its larger particle size (P.S), providing additional space for saponin accommodation and enhanced sequestration within the vesicles [54]. Notably, the EE% of the nanocomposites remained stable over 3 months of storage (P > 0.05), reflecting the robust encapsulation properties of the formulations, consistent with findings by Manca et al. [55]. Furthermore, several reports demonstrated the chemical stability of both saponins owing to their distinct molecular structure, which consists of a hydrophobic aglycone (sapogenin) and hydrophilic sugar moieties. Saponins' amphiphilic nature enables them to remain stable in a variety of environments, including aqueous and lipid-based systems. Meanwhile, the glycosidic linkages that link the sugar units to the sapogenin core are generally resistant to hydrolysis under normal physiological conditions, which increases their stability. The presence of numerous hydroxyl groups in their structure also helps them develop stable interactions with other molecules such as lipids and proteins, which can protect them against degradation [48]. Moreover, encapsulating saponins in nanoparticles increased their chemical stability and prevented degradation. Nanoparticles form a protective barrier around drug molecules, shielding them from environmental conditions including light, heat, oxygen, and moisture that can cause chemical degradation. This encapsulation also prevents premature interactions with enzymes or other reactive chemicals in the body, ensuring that the drug remains intact until it reaches its targeted site of action. Furthermore, nanoparticles' controlled release capabilities ensure that the drug is released in a stable and prolonged manner, lowering the possibility of degradation over time [45].

The sustained release of saponin(s) observed in the release profile of all the prepared vesicles could be ascribed to the enhanced saponin(s) encapsulation within the vesicular systems owing to their aforementioned lipophilicity, promoting the sustainment of their diffusion throughout the vesicular structures [56].

In the present study, PEVIII demonstrated significant antimicrobial and anti-virulent activity in vitro, with a minimum inhibitory concentration (MIC) of 64 μg/mL. Furthermore, PEVIII resulted in a substantial reduction in key virulence factors associated with the induction of keratitis, including a 50% decrease in pyocyanin production and an 80% reduction in twitching motility. These findings highlight the potential of PEVIII as an effective therapeutic agent against *P. aeruginosa*-induced keratitis. Several studies reported that twitching motility is essential for in vivo corneal infection induction to aid bacterial transfer from epithelium to underlying stroma [7]. The mechanisms of PEVIII antimicrobial properties may be attributed to the enhanced bacterial cell wall penetration, which is aided by its lipophilic character as well as immune system stimulation [57].

The histological findings from this study provide valuable insights into the efficacy of different therapeutic interventions for *P. aeruginosa*-induced keratitis. The untreated infected groups demonstrated severe disruption of corneal tissue architecture, characterized by epithelial loss, extensive inflammatory cell infiltration, and stromal neovascularization. These pathological changes highlight the aggressive nature of *P. aeruginosa*-induced keratitis and the urgent need for effective therapeutic strategies. PEVI and PEVII treatments showed partial improvements, suggesting a degree of efficacy in mitigating inflammatory responses and tissue damage. This partial improvement might be attributed to the individual therapeutic properties of the encapsulated saponins, which may not have been sufficient to completely counteract the aggressive pathogenesis of the infection. In contrast, PEVIII treatment resulted in pronounced histological improvement, as evidenced by the preservation of the corneal epithelial lining, minimal stromal edema, and the absence of inflammatory cell infiltration. These results indicate that the co-encapsulation of both α-hederin and hederacoside C in PEVIII synergistically enhances therapeutic efficacy. The improved encapsulation efficiency and increased particle size in PEVIII likely contributed to prolonged drug release, enhanced bioavailability, and targeted delivery, leading to better therapeutic outcomes. The superior performance of PEVIII compared to GENTAWISE®, a widely used antibiotic formulation, further underscores the potential advantages of nanocomposite-based delivery systems. To further validate the anti-inflammatory effects of saponin treatments, we estimated the extent of proinflammatory cytokines expression levels (TNF-α and NF-κB), ROS levels, AKT1 and PI3K, OprL expression levels in corneas infected with *P. aeruginosa.* The NF-κB signaling pathway plays a pivotal role in regulating inflammatory responses, particularly through tumor necrosis factor-alpha (TNF-α) expression [58]. Saponins directly inhibit NF-κB activity, leading to a reduction in TNF-α production, reducing inflammation, and accelerating the corneal healing process [59, 60]. PEVIII demonstrated the most significant therapeutic efficacy in the treatment of *P. aeruginosa* keratitis. It led to a substantial reduction in inflammatory markers, including a 68.8% decrease in TNF-α and a 62% reduction in NF-κB, surpassing the effects of other treatments, including GENTAWISE^®^. These findings support the potential of saponins in attenuating the inflammatory cascade associated with Pseudomonas keratitis, as observed in the current study.

One of the key mechanisms by which saponins exert their protective effects is through the reduction of reactive oxygen species (ROS) levels [61, 62]. By reducing ROS, saponins alleviate oxidative stress and its associated cellular damage, especially relevant in conditions like Pseudomonas keratitis. In this study, PEVIII exhibited a significant reduction in ROS by 73.3%, indicating enhanced antioxidant capacity. The PI3K/Akt signaling pathway plays a central role in maintaining cellular redox homeostasis through activation of antioxidant responses. Increased activity of this pathway is known to upregulate key antioxidant enzymes such as superoxide dismutase and catalase, facilitating ROS detoxification and protecting cells from oxidative stress. In line with this, we measured both ROS and PI3K/Akt levels to assess the scavenging potential. PEVIII displayed the highest activation of protective signaling pathways, with a 5.75-fold increase in AKT1 and a 4.97-fold increase in PI3K, correlating with a significant reduction in ROS. This suggests that the enhanced antioxidant response is at least partially mediated by PI3K/Akt signaling. This finding aligns with previous researchs, including Wang et al. [63] and Wen et al. [64] which highlighted the role of PI3K/Akt activation in promoting redox balance and protecting cell from oxidative damage [63, 64]. Additionally, saponins are reported to stimulate the PI3K/Akt signaling pathway, contributing not only to antioxidant effects but also to improved cell growth, survival, and anti-inflammatory responses [65]. Our results align with the work of Wijesekara et al. [66], demonstrating that saponins enhance Akt production and subsequently suppress the release of pro-inflammatory cytokines [66]. Altogether, these findings suggest that saponins promote tissue healing in PA keratitis through a combined antioxidant and anti-inflammatory mechanism involving PI3K/Akt pathway activation. The outer membrane lipoprotein OprL is a significant virulence factor in *P. aeruginosa*, contributing to the bacterium's pathogenicity through various mechanisms. OprL is involved in the synthesis of lipopolysaccharide (LPS), a critical component of the bacterial outer membrane that plays a vital role in motility, colonization of host tissues, and the invasion of bacterial active proteins into target cells [44, 67]. By reducing OprL expression, saponins effectively lower bacterial virulence and enhance host immune responses. Interestingly, PEVIII treatment demonstrated the strongest reduction in the virulence factor OprL by 79.2% and emerged as the most effective treatment in mitigating the virulence factor OprL, demonstrating its potential for better control of bacterial virulence in Pseudomonas keratitis. Overall, PEVIII proved to be the most effective treatment, enhancing tissue protection, reducing inflammation, and controlling bacterial virulence, highlighting its potential as a leading therapeutic option for *P. aeruginosa* keratitis.

Saponins are a structurally diverse class of amphiphilic glycosides that have garnered increasing interest in ophthalmic drug delivery due to their multifaceted pharmacological properties [68]. Their potential therapeutic benefits in ocular diseases stem from their anti-inflammatory**,** antioxidant, and immunomodulatory effects. For instance, glycyrrhizin, a triterpenoid saponin, has proven substantial efficacy in ocular treatment of *P. aeruginosa* Keratitis [69], corneal neovascularization [70], and dry eye disease [71] with good tolerability. Similarly, the ocular application of ginsenosides Rg1 and R_b_1 protect against diabetic retinopathy [72, 73], ginsenoside Rh2 attenuates corneal neovascularization [74], and ginsenoside Rg3 safeguards against Age-related Macular Degeneration (AMD) [75]. Despite these promising effects, the clinical translation of saponins for ocular use is limited due to their inherent surfactant-like properties, which can activate nociceptor TRPV1 channels, leading to ocular irritation or discomfort [76]. To overcome these limitations, several formulation strategies have been proposed. For example, nanoencapsulation within biocompatible carriers such as liposomes, solid lipid nanoparticles, or chitosan-coated vesicles can reduce direct contact of saponins with ocular tissues, modulate their release profile and enhance their bioavailability and therapeutic efficacy [77, 78]. In this study, α-hederin and hederacoside C were successfully encapsulated within chitosan-coated penetration enhancer vesicles (PEVs) to improve their ocular delivery. The chitosan's mucoadhesive properties might prolong their corneal residence time and create a protective barrier between the saponins and the ocular surface [79]. Moreover, the anti-inflammatory, antibacterial, wound-healing, and immunomodulatory properties of chitosan further provide ocular surface protection, which in turn reduces the risk of irritation or inflammation that might be triggered by the surfactant-like nature of saponins [80–82]. Additionally, the in vivo study revealed no signs of ocular discomforts, such as excessive blinking, redness, or tearing during or after the 7-day treatment. This was further supported by histopathological analysis, which confirmed that none of the PEV formulations caused epithelial damage or elicited an inflammatory response. Notably, the group treated with the saponin-loaded PEVIII formulation exhibited remarkable tissue regeneration and significant reduction in inflammatory markers, underscoring the safety and therapeutic potential of the PEVIII formulation.

## Conclusion

Hedera saponins have demonstrated significant therapeutic potential in managing Pseudomonas keratitis. Their multifaceted benefits include reducing oxidative stress, modulating inflammatory pathways, and targeting bacterial virulence factors. Encapsulation of saponins into composite nano vesicular systems has shown enhanced antimicrobial, anti-virulent, antioxidant, and anti-inflammatory activities, posing them as promising candidates for topical ocular applications. Consequently, the topical administration of PEVIII represents a promising therapeutic strategy for combating Pseudomonas-induced keratitis. Although the sample size in this study was relatively small (n = 5 per group), we acknowledge that this limitation could influence the generalizability of our findings. The chosen sample size was based on ethical considerations, experimental feasibility, and was consistent with prior studies in similar keratitis models. For instance, Ren and Wu [83] used a comparable sample size in their neutropenic mouse model while demonstrating clear treatment efficacy [83]. Despite the small sample size, we reported large effect sizes, which strongly support the biological relevance of our findings. To mitigate bias, randomization was employed based on baseline health status, and histopathological assessments were conducted by an independent pathologist blinded to group allocations. Given the exploratory nature of our study and its alignment with established models, we consider our methodology to be robust. However, we acknowledge that larger sample sizes would be beneficial in future studies to validate and expand upon our findings. Further futuristic studies using animal tissues and clinical research are recommended to investigate the ex vivo corneal permeation and the pharmacokinetics of these nanosystems to optimize their efficacy and applicability to human health.

## Supplementary Information

Supplementary material 1.

## Data Availability

Data available within the article or its supplementary materials.
