# Supplementary material for: Therapeutic targeting of ocular diseases with emphasis on PI3K/Akt, and OPRL pathways by Hedera helix L. saponins: a new approach for the treatment of Pseudomonas aeruginosa-induced bacterial keratitis
Source: Nat Prod Bioprospect. 2025 May 12;15(1):30. doi: 10.1007/s13659-025-00514-x (PMC12069210; doi:10.1007/s13659-025-00514-x)
Supplement: Supplementary file 1 — Supplementary material 1. [file 13659_2025_514_MOESM1_ESM.pdf]

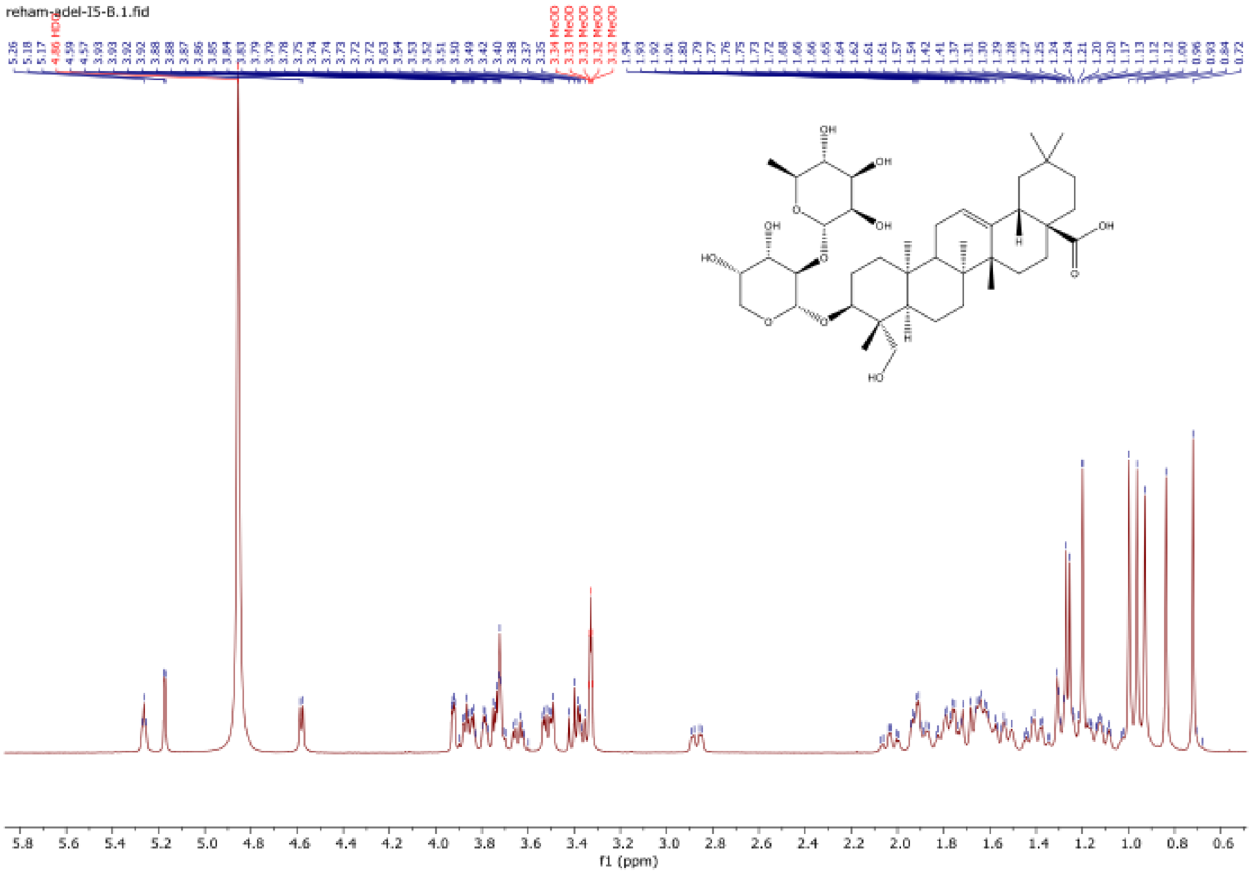


**Fig. S1.** $^{1}$HNMR of α-hederin.

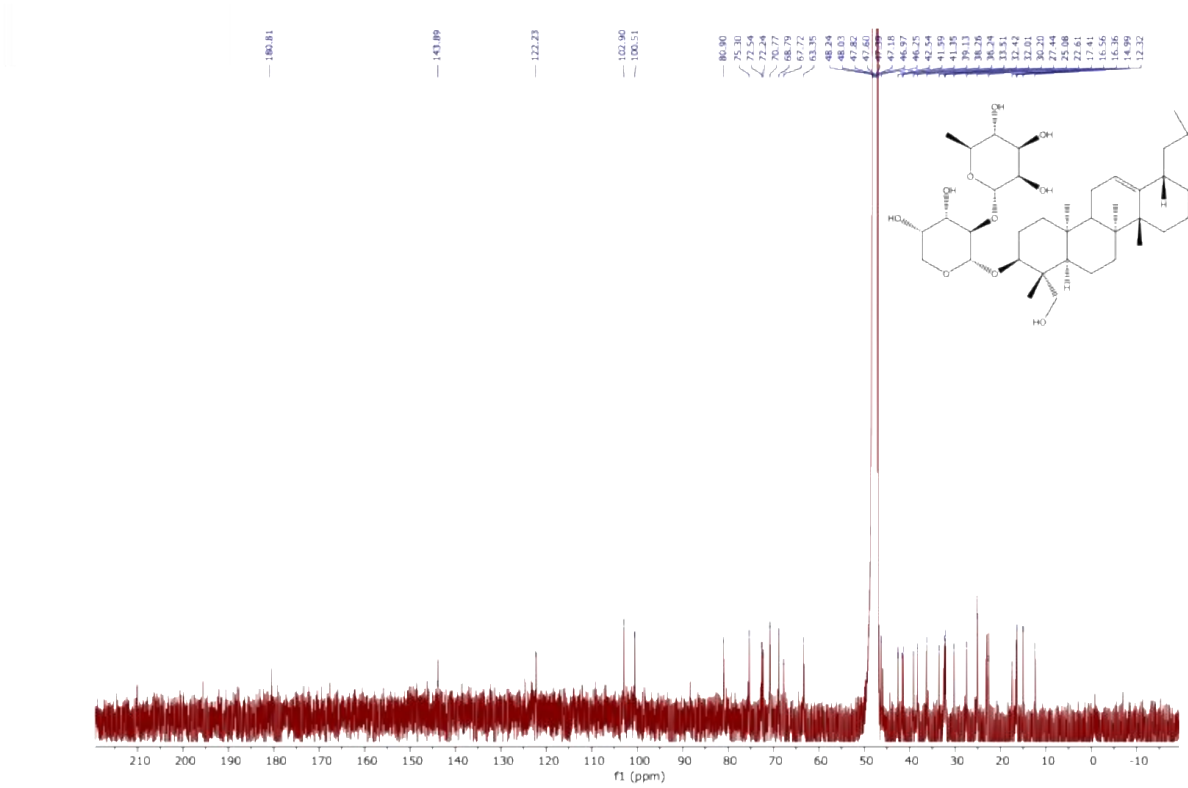

**Fig. S2.** $^{13}$CNMR of α-hederin.

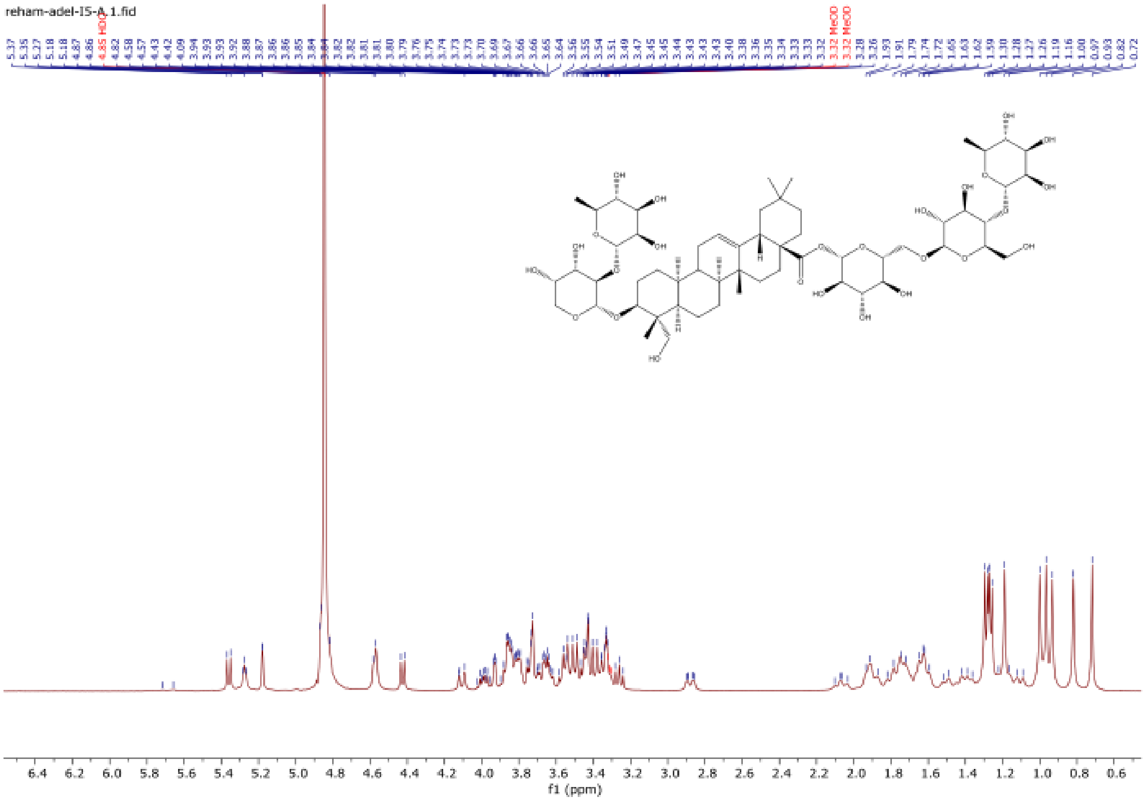


**Fig. S3.** $^{1}$HNMR of hederacoside C.

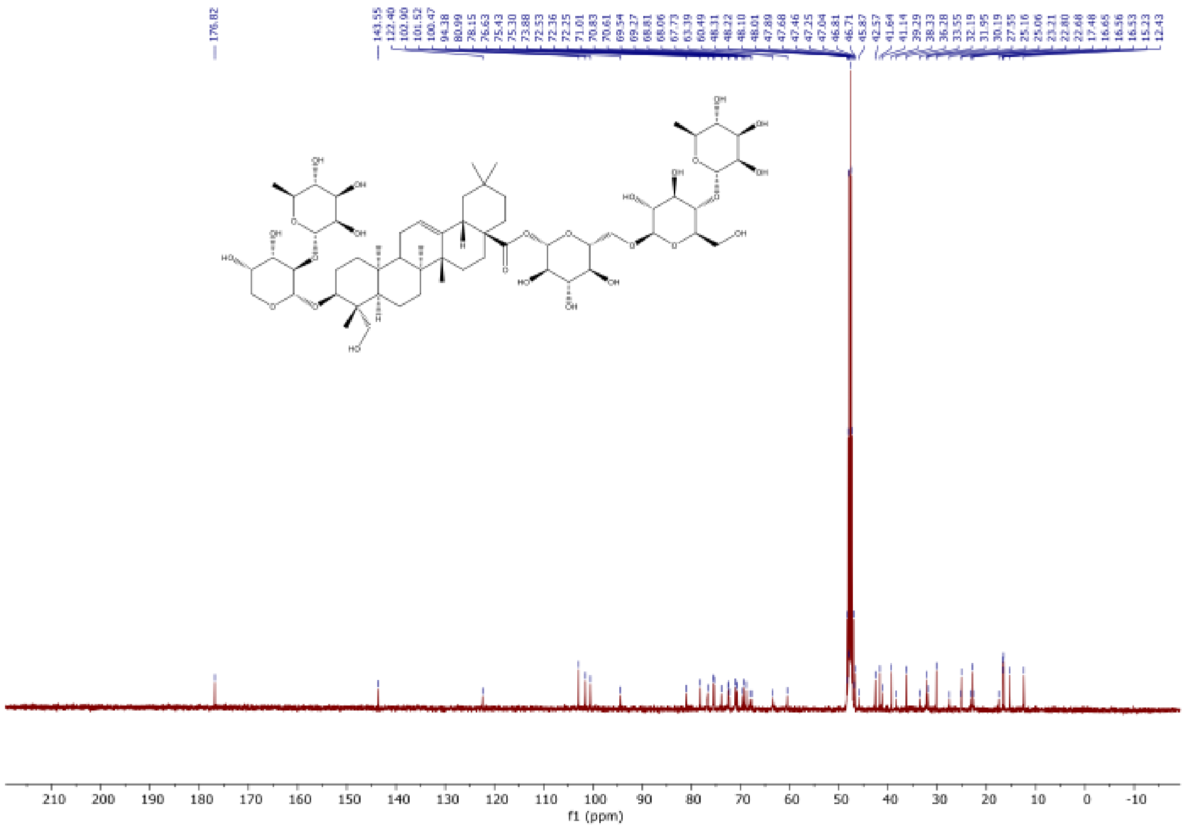


**Fig. S4.** $^{13}$CNMR of hederacoside C.

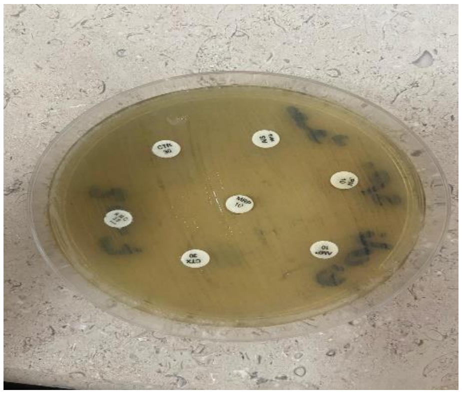


**Fig. S5.** Carbapenem resistant *Pseudomonas aeruginosa* (CRPA).
